# Amos Bairoch (1957–2025): pioneer of bioinformatics and founder of Swiss-Prot

**DOI:** 10.1093/bioadv/vbag009

**Published:** 2026-02-06

**Authors:** Alex Bateman

**Affiliations:** European Molecular Biology Laboratory, European Bioinformatics Institute (EMBL-EBI), Wellcome Genome Campus, Hinxton CB10 1SD, United Kingdom

Amos Bairoch, one of the founding figures of bioinformatics, passed away on 1 December 2025 at the age of 68 (SIB Swiss Institute of Bioinformatics 2025a). As the creator of Swiss-Prot, PROSITE, and numerous other resources that form the bedrock of modern protein bioinformatics, Amos’ contributions have shaped how scientists worldwide access, organize, and understand biological data (Wikipedia 2025, Wiper 2025).

**Figure:**
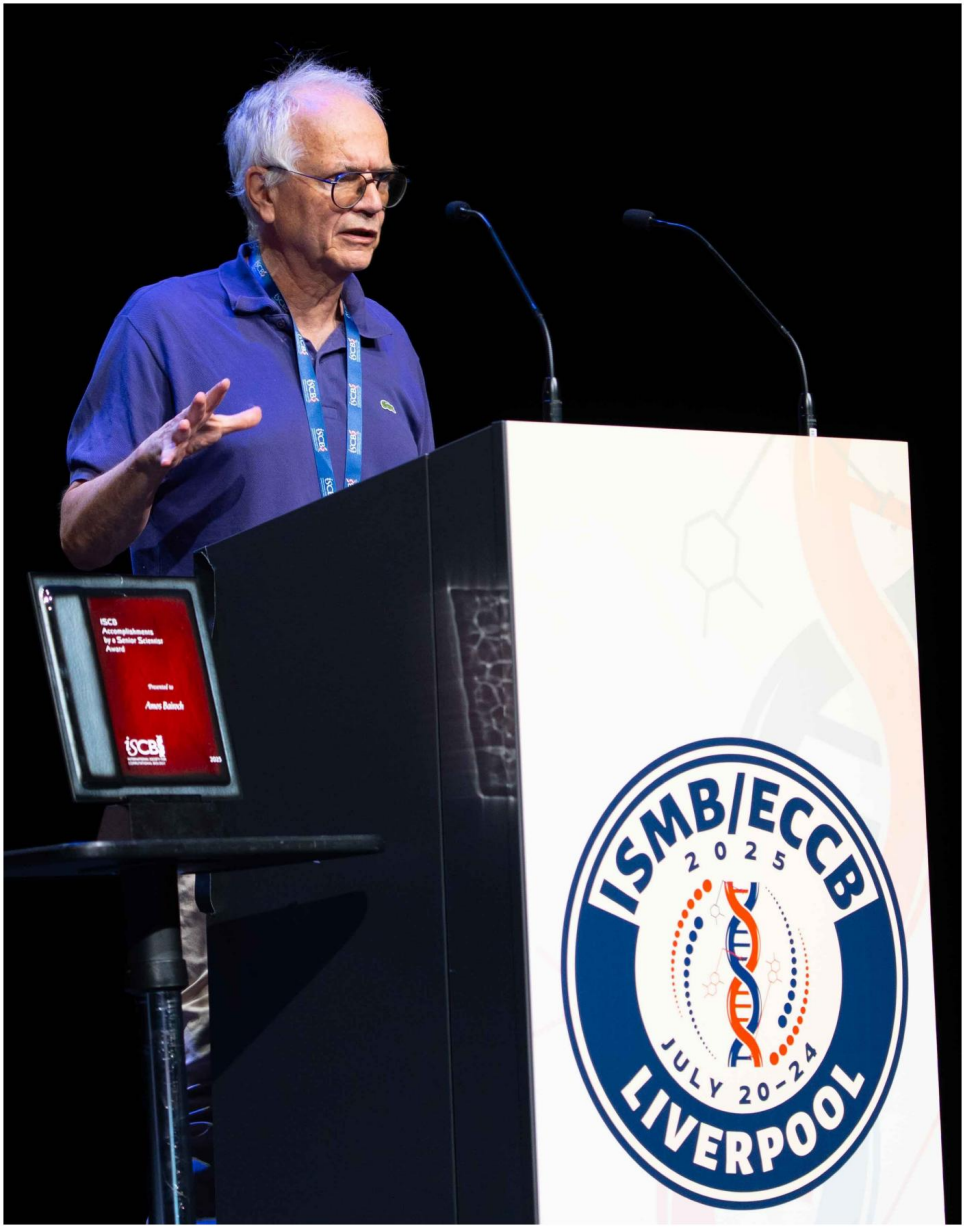
Amos giving his keynote presentation at ISMB 2025. Photo credit: Michaëlle Jade Lanoue. Copyright International Society for Computational Biology (2025).

## Early life and the path to bioinformatics

Born on 22 November 1957 in Geneva, Switzerland (Wikipedia 2025), Amos’ scientific curiosity was sparked early when his father, the economic historian Paul Bairoch, gave him a book on astronomy (Wiper 2025). Though initially drawn to space exploration and exobiology—fields that were not yet established in European academia at the time—he pivoted to the fields of biochemistry and mathematics, believing these to be the foundation for understanding life’s chemical basis (Wiper 2025).

His entry into computational biology came through an unexpected route: a TRS-80 computer his father purchased for statistical analyses. Amos began writing programs to assist with these analyses, and soon discovered papers from the laboratories of Rodger Staden and Rich Roberts describing tools for sequence analysis on mainframe computers (Wiper 2025). He saw a different possibility—bringing these capabilities to personal computers, making sequence analysis accessible to individual laboratories rather than restricted to major institutions (SIB Swiss Institute of Bioinformatics 2025b, Wiper 2025).

## PC/gene and the birth of Swiss-Prot

Amos brought his vision to Dr Robin Offord, a protein chemist at the University of Geneva, who agreed to supervise his master’s thesis and subsequently his PhD. With Dr Jean-Michel Claverie at the Pasteur Institute as co-advisor (Wiper 2025), Amos developed PC/Gene, an MS-DOS-based software package for protein and nucleotide sequence analysis. The software was commercialized first by Swiss company Genofit, then by IntelliGenetics in the USA, which was later acquired by Oxford Molecular (Wikipedia 2025).

It was during this work that Amos began developing what would become his most significant contribution: an annotated protein sequence database. Swiss-Prot was first released in July 1986 with nearly 4000 curated protein sequence entries (SIB Swiss Institute of Bioinformatics 2025b, Wikipedia 2025). Though not part of the original PhD plan, the database became the centerpiece of his work, ultimately delaying his degree completion—a delay his supportive advisors fully endorsed (Wiper 2025).

## Building international infrastructure

From 1987, Swiss-Prot became a collaborative project with the Data Library group at the European Molecular Biology Laboratory, which later evolved into the European Bioinformatics Institute (EBI) in the UK (Wikipedia 2025). This European partnership proved transformative.

Rolf Apweiler, now Director Emeritus of EMBL-EBI, recalls how his career began with that collaboration: “After founding Swiss-Prot in 1986, Amos reached out to EMBL Heidelberg the following year, where Patricia Kahn and Graham Cameron hired me as a student helper for this new and exciting project. For 7 years I worked mostly part-time as a Swiss-Prot curator, exchanging countless emails with Amos and seeing him only during his occasional visits to EMBL. I learned an enormous amount from him.”

In 1994, Apweiler became Head of the Swiss-Prot group at EMBL-EBI, and over the following decade, the two developed a productive division of labor. “He devoted himself passionately to meticulous manual curation, while I focused on people management, infrastructure, modern data management, automation, standardisation, and securing funding,” Apweiler explains. “Our discussions were often intense, sometimes controversial, but always fruitful. The balance we forged between quality, scale, and reliability helped shape these resources into cornerstones of modern biological data infrastructure.”

In 2002, the collaboration expanded across the Atlantic with the creation of UniProt—the Universal Protein Resource—joining Swiss-Prot and TrEMBL with the American Protein Information Resource (PIR) (SIB Swiss Institute of Bioinformatics 2025b, Wikipedia 2025). Cathy Wu, Director of PIR at the University of Delaware, remembers how this partnership began: “Amos’ untimely passing brought back memories of how we first met and discussed annotation errors in databases at the Mathematical Modelling and Scientific Computing conference in Washington DC, 1997. That later led to our partnership in establishing the UniProt Consortium along with Rolf that brought together the SIB, EBI and PIR teams in 2002.”

Today, UniProt stands as the world’s most comprehensive catalogue of protein sequence and function information, a direct descendant of Amos’ original vision.

## PROSITE and the classification of protein families

Swiss-Prot was far from Amos’ only contribution. In 1988, he created PROSITE, a database of protein families and domains that enables the identification of protein functions through pattern and profile matching (SIB Swiss Institute of Bioinformatics 2025b, Wikipedia 2025). PROSITE provided one of the earliest systematic approaches to classifying proteins into families based on conserved sequence motifs, enabling researchers to infer function for newly sequenced proteins.

PROSITE later became a founding member of the InterPro consortium, which integrates protein family and domain information from multiple databases to provide a unified resource for protein classification and functional annotation.

I first met Amos at the Aron Katzir-Katchalsky conference in Jerusalem in the mid-1990s. As a PhD student working on protein family analysis, I had provided feedback on immunoglobulin and fibronectin type III domain annotations in Swiss-Prot. During his keynote lecture at the conference, Amos mentioned me as a “Swiss-Prot super user”—a recognition that motivated me to continue the work in protein family classification that has shaped my career. Amos’ drive to capture everything known about proteins seemed like an impossible task, but his enthusiasm knew no bounds.

## ExPASy and ENZYME

The ENZYME database followed PROSITE in 1990, providing systematic nomenclature for enzymes that became indispensable for the development of metabolic databases (SIB Swiss Institute of Bioinformatics 2025b).

In August 1993, together with Ron Appel, Amos launched ExPASy—the Expert Protein Analysis System (Wikipedia 2025). This was the first molecular biology World Wide Web server, and among the first 150 websites in existence (SIB Swiss Institute of Bioinformatics 2025b). What began as a prototype rapidly grew into a major portal providing access to databases and analysis tools for proteomics research.

Francis Ouellette remembers his first encounter with ExPASy in 1993, when Amos visited his boss Mark Boguski at NCBI: “‘Bonjour, Francis, as-tu vu mon site web de Swiss-Prot?’ On my Netscape browser he typed: EXPASY.CH. It was the first bioinformatics website I ever visited on a WWW browser!”

## Founding the Swiss Institute of Bioinformatics

Recognizing that bioinformatics required dedicated institutional support, Amos co-founded the Swiss Institute of Bioinformatics (SIB) in 1998 with colleagues in Geneva and Lausanne (Wikipedia 2025, Wiper 2025). The institute was designed to support the long-term stability of bioinformatics research in Switzerland, with an emphasis on research, education, services, and the development of databases and tools (Wikipedia 2025).

Ioannis Xenarios, former UniProt principal investigator at SIB, recalls how Amos supported him from the very beginning of his career: “When I was a master student and my family could not really make ends meet, Amos suggested that I could do curation on Swiss-Prot as a way to help—he had that level of support for people. He has been the only reason why I left Pharma/biotech to join the SIB.”

In November 1997, together with Ron Appel and Denis Hochstrasser, Amos founded GeneBio (Geneva Bioinformatics SA). In April 2000, with Keith Rose and Robin Offord, he co-founded GeneProt, a high-throughput proteomics company that operated until 2005 (Wikipedia 2025).

## neXtProt, Cellosaurus, and continued innovation

From 2009, Amos led the CALIPHO (Computer Analysis and Laboratory Investigation of Proteins of Human Origin) group at SIB, jointly with Lydie Lane (SIB Swiss Institute of Bioinformatics 2025c, Wikipedia 2025). The group focused on neXtProt, a comprehensive knowledge platform on human proteins that complements UniProtKB/Swiss-Prot with data from proteomics, transcriptomics, and genomics (SIB Swiss Institute of Bioinformatics 2025c).

Lane, who first joined Swiss-Prot as a curator in 2004, recalls: “What struck me most was his attention to detail—not as perfectionism, but as respect for data, for users, and for the collective effort of the scientific community. He carefully reviewed every line of Swiss-Prot with kindness, turning verification into a shared act of trust and learning.”

Working side by side on the CALIPHO/neXtProt adventure, Lane came to appreciate “his unwavering optimism, boundless curiosity, and the genuine joy he took in collaborating with people from diverse fields. These interactions sparked countless ideas, the brightest of which was the Cellosaurus—a thesaurus of cell lines created to support experimental research.”

The Cellosaurus (SIB Swiss Institute of Bioinformatics 2025a, 2025c) has become an essential tool for researchers worldwide. Amos’ attention extended to the often-overlooked issue of cell line misidentification and contamination, helping to improve reproducibility in biomedical research.

Ruedi Aebersold of ETH Zurich met Amos at an event in Zurich <2 weeks before his death, where Amos was talking with his usual enthusiasm about his plans. Aebersold recalls the early days: “Going back to the first Siena meetings I see him sitting towards the back of the lecture hall, laptop open and typing in any new information presented in real time into his database. Who would have thought at the time what these early steps would evolve into, supporting the work of generations of biologists, clinical scientists and informaticians.”

## Founding the profession of biocuration

Beyond database development, Amos was a founder member of the field of biocuration and played a fundamental role in it becoming a recognized profession, not least through his input into the establishment of the International Society for Biocuration. He trained biocurators before formal or standardized training programs existed (SIB Swiss Institute of Bioinformatics 2025b, Wiper 2025), establishing standards and practices that continue to guide the field.

Sandra Orchard, Team Leader for Protein Function Content at EMBL-EBI, describes his lasting influence: “His depth of biomedical knowledge and understanding of protein function resulted in him being a force to be reckoned with in any discussion—he could always pull up the relevant UniProtKB/Swiss-Prot entry from memory to prove his point. His attention to detail and clearly defined curation rules were copied and adapted by many biological databases and he inspired generations of biocurators and bioinformaticians to develop data standards, ontologies and tools for data capture and analysis. It is a tribute to Amos’s depth of vision that the curation rules he started to develop almost 40 years ago for the first Swiss-Prot entries still form the basis of the work of the UniProtKB staff today.”

Cecilia Arighi of the University of Delaware agrees: “Amos and biocuration are synonyms. He was the most dedicated and enthusiastic biocurator I’ve ever known. He really believed in the field and nurtured so many of us to follow it. I had the honor to spend a few weeks in Geneva to learn about Swiss-Prot annotation when I started in UniProt, and he was so welcoming. I will always remember his smile when passionately explaining some interesting protein annotation!”

Peer Bork, Director at EMBL Heidelberg, recalls discussions from the early 1990s about the potential of sequence data: “We then already emphasized the need for high quality data, which is essential for any AI work these days. Amos himself was a walking knowledge base.”

## Recognition and honours

Amos’ contributions were recognized with numerous awards throughout his career: the Friedrich Miescher Award from the Swiss Society of Biochemistry (1993), the Helmut Horten Foundation Incentive Award (1995), the Pehr Edman Award (2004), the European Latsis Prize (2004), the Otto Naegeli Prize (2010), the HUPO Distinguished Achievement Award in Proteomic Sciences (2011), the EUPA Proteomics Pioneer Award (2013), and the ABRF Award (2018) (Wikipedia 2025).

In July 2025, just months before his death, Amos received the ISCB Accomplishments by a Senior Scientist Award at the joint ISMB/ECCB conference in Liverpool (SIB Swiss Institute of Bioinformatics 2025b, Wiper 2025). He delivered a keynote lecture reflecting on 40 years of biocuration (SIB Swiss Institute of Bioinformatics 2025b)—a fitting capstone to an extraordinary career.

## Legacy

The resources Amos created—Swiss-Prot, UniProt, PROSITE, ENZYME, ExPASy, neXtProt, Cellosaurus—continue to underpin biological research worldwide. His commitment to open access, high-quality curation, and the integration of human expertise with computational approaches established principles that guide bioinformatics to this day (SIB Swiss Institute of Bioinformatics 2025a, 2025b).

But colleagues remember more than his scientific contributions. Maria Martin, Team Leader for UniProt Content at EMBL-EBI, recalls: “Amos cared enormously for those he worked with, embracing them as though they were family. His passion for curation and Swiss-Prot shone through in everything he did, and he never missed a chance to share joy and celebrate the moments that mattered. He organized memorable group retreats in interesting places with excellent food, something he took great pride in.” One such celebration was the 20th anniversary of Swiss-Prot in Fortaleza, where, true to form, “he was the first one dancing.”

Lydie Lane reflects on what she learned from him: “Amos had a rare talent for building teams around shared values, fostering collaboration without hierarchy, and mentoring by example. Observing his approach shaped my own standards: rigor paired with humility, and ambition anchored in service rather than visibility. His impact is quiet, durable, profoundly human, and will live on in both his resources and the generations of scientists he inspired.”

Richard Durbin of the University of Cambridge summarizes the qualities that made Amos exceptional: “Like so many of you, my core memories of Amos are of him editing SwissProt in real time. Knowledge, commitment, judgement and courage.”

Christos Ouzounis of Aristotle University of Thessaloniki, who first met Amos in 1989 at EMBL, reflects: “Ever since we shared wonderful interactions, always intellectually stimulating, warm, and collegial for so long. He was truly one-of-a-kind. Our tiny microcosm will never be the same without him.”

“Working with Amos for 20 years was not always easy, but it was profoundly rewarding,” concludes Rolf Apweiler. “His energy, his deep knowledge, and his humour will be greatly missed.”
